# Association of Hepatocyte Growth Factor and Angiopoietin-2 with Systemic Cardiovascular Risk in Patients with Peripheral Artery Disease

**DOI:** 10.3390/jcm14124031

**Published:** 2025-06-06

**Authors:** Ben Li, Adam M. Khalil, Lina Abuhalimeh, Farah Shaikh, Houssam Younes, Batool Abuhalimeh, Abdelrahman Zamzam, Rawand Abdin, Mohammad Qadura

**Affiliations:** 1 Department of Surgery, University of Toronto, Toronto, ON M5S 1A1, Canada; benx.li@mail.utoronto.ca; 2 Division of Vascular Surgery, St. Michael’s Hospital, Unity Health Toronto, University of Toronto, Toronto, ON M5B 1W8, Canada; 3 Institute of Medical Science, University of Toronto, Toronto, ON M5S 1A1, Canada; 4 Temerty Centre for Artificial Intelligence Research and Education in Medicine (T-CAIREM), University of Toronto, Toronto, ON M5S 1A1, Canada; 5 Medical College, Weill Cornell Medicine, Ar-Rayyan 8C9R+735, Qatar; 6 College of Medicine, University of Jordan, Amman 11942, Jordan; 7 Heart, Vascular, & Thoracic Institute, Cleveland Clinic Abu Dhabi, Abu Dhabi 112412, United Arab Emirates; 8 Department of Medicine, McMaster University, Hamilton, ON L8S 4L8, Canada; 9 Li Ka Shing Knowledge Institute, St. Michael’s Hospital, Unity Health Toronto, University of Toronto, Toronto, ON M5B 1W8, Canada

**Keywords:** angiogenesis-related proteins, hepatocyte growth factor, angiopoietin-2, major adverse cardiovascular events, prognosis, peripheral artery disease

## Abstract

**Background/Objectives:** Major adverse cardiovascular events (MACE) are the primary cause of mortality among individuals with peripheral artery disease (PAD). Despite this, there is limited research on biomarkers that can predict MACE risk in this population. Proteins involved in angiogenesis are integral to both systemic circulation and the development of atherosclerosis, indicating their potential as prognostic markers. This study aimed to identify angiogenesis-related proteins associated with MACE risk in PAD patients. **Methods:** We conducted a prospective cohort study involving 250 patients diagnosed with PAD. At baseline, plasma levels of 17 angiogenesis-related proteins were measured. Participants were followed for two years, with the primary outcome being the incidence of MACE—a composite of stroke, myocardial infarction, or death. Protein concentrations were compared between those who experienced 2-year MACE and those who did not using the Mann–Whitney U test. Proteins showing significant differences were further analyzed using Cox proportional hazards modeling to assess their independent associations with MACE, adjusting for baseline demographic and clinical variables, including prior coronary and cerebrovascular disease. Kaplan–Meier survival analysis was also employed to compare MACE-free survival based on protein concentration levels. **Results:** The average age of participants was 69 years (SD 9), with 32% (*n* = 80) being female. Over the two-year follow-up, 48 patients (19.8%) experienced MACE. Among the proteins assessed, only hepatocyte growth factor (HGF) and angiopoietin-2 were significantly elevated in patients who developed MACE (HGF: 390.83 [SD 319.16] vs. 300.55 [SD 177.53] pg/mL, *p* < 0.001; angiopoietin-2: 23.67 [SD 17.60] vs. 19.36 [SD 12.06] pg/mL, *p* = 0.020). Multivariable Cox analysis confirmed that elevated levels of both HGF (adjusted HR 1.37; 95% CI 1.14–1.64; *p* = 0.001) and angiopoietin-2 (adjusted HR 1.27; 95% CI 1.04–1.55; *p* = 0.016) were independently associated with increased 2-year MACE risk. Kaplan–Meier curves demonstrated significantly reduced MACE-free survival in patients with higher levels of HGF and angiopoietin-2. **Conclusions:** HGF and angiopoietin-2 emerged as significant, independent predictors of 2-year MACE in patients with PAD. Measuring plasma levels of these proteins may enhance risk stratification, guiding referrals to appropriate cardiovascular specialists and informing the intensity of medical management. This biomarker-based precision medicine approach holds potential for improving cardiovascular outcomes in the PAD population.

## 1. Introduction

Peripheral artery disease (PAD), defined by atherosclerotic obstruction in the lower extremity arteries, affects more than 200 million people worldwide [1]. Although PAD is commonly linked to major adverse limb events (MALE) including amputation, most patients ultimately die from major adverse cardiovascular events (MACE), including myocardial infarction (MI) and stroke [2]. This increased cardiovascular risk is primarily attributed to the strong overlap between PAD and other forms of systemic atherosclerosis, particularly coronary artery disease (CAD) and cerebrovascular disease (CVD) [3]. Shared risk factors—such as aging, hypertension, diabetes, dyslipidemia, and tobacco use—contribute to the widespread vascular involvement seen in PAD patients [4]. Given this systemic nature, early identification of PAD patients at heightened risk of MACE is critical. Accurate risk stratification allows for timely multidisciplinary intervention and the implementation of aggressive cardiovascular risk reduction strategies [5]. One promising avenue for improving risk prediction is the discovery of novel circulating biomarkers [6,7,8]. While our research group has previously identified angiogenic biomarkers associated with MALE [9], the investigation of biomarkers predictive of broader cardiovascular outcomes in the PAD population remains limited and underexplored.

Angiogenesis—the formation of new blood vessels—is a critical physiological response to vascular injury, particularly in ischemic conditions associated with atherosclerosis [10]. Angiogenesis-related proteins, such as hepatocyte growth factor (HGF) and angiopoietin-2, play vital roles in endothelial function, tissue perfusion, and atherosclerotic plaque dynamics, and have been implicated in various vascular pathologies, including PAD, CAD, and CVD [11,12]. Over 15 such proteins have been linked to these conditions, supporting their relevance in the broader context of systemic vascular disease [13,14,15,16]. Due to their role in vascular biology and systemic atherosclerosis, angiogenesis-related proteins may hold promise as biomarkers for early MACE detection in patients with PAD [10]. The 17 proteins selected for analysis in this study were chosen based on substantial prior research demonstrating their association with cardiovascular disease and their potential predictive utility for systemic cardiovascular risk in PAD patients [13,14,15,16]. Although several studies have reported associations between these proteins and cardiovascular conditions, few have specifically evaluated their prognostic significance for MACE in the PAD population. Previously, our group showed that angiogenesis-related proteins are associated with MALE in PAD patients [9]. However, their ability to predict MACE has not been thoroughly examined. This study aims to fill that gap by identifying angiogenesis-related biomarkers with prognostic value for MACE in the PAD population, with the broader goal of improving early risk stratification and guiding intensive treatment strategies for patients with systemic atherosclerosis.

## 2. Materials and Methods

### 2.1. Ethical Approval

The Research Ethics Board at Unity Health Toronto granted ethical clearance for this study on 8 February 2017 (REB #16-375). Informed consent was obtained from all participants prior to enrollment. All study procedures adhered strictly to the ethical standards outlined in the Declaration of Helsinki [17].

### 2.2. Study Design

This research was structured as a prognostic investigation, and its results were presented following the Transparent Reporting of a multivariable prediction model for Individual Prognosis Or Diagnosis + Artificial Intelligence guidelines [18].

### 2.3. Patient Recruitment

From January 2018 to August 2019, consecutive patients with PAD were prospectively enrolled from the ambulatory vascular clinics at St. Michael’s Hospital. PAD was diagnosed using an Ankle-Brachial Index (ABI) below 0.9 or a Toe-Brachial Index (TBI) below 0.7, along with the presence of weak or absent pedal pulses [19]. Individuals were not included in the study if they had suffered from acute limb ischemia or acute coronary syndrome, or had elevated troponin levels in the three months prior to assessment. The study cohort comprised 250 individuals diagnosed with PAD.

### 2.4. Baseline Patient Characteristics

The study documented participants’ baseline demographics and clinical profiles, including age, sex, smoking status (both current and former), and pre-existing conditions including hypertension, diabetes, dyslipidemia, congestive heart failure (CHF), CAD, and prior stroke. Cardiovascular risk factors were classified based on criteria established by the American College of Cardiology [20,21]. Hypertension was characterized by a diastolic pressure of at least 80 mmHg, a systolic blood pressure of 130 mmHg or greater, or the use of antihypertensive therapy [20,21]. Dyslipidemia was identified by triglyceride levels above 1.7 mmol/L, a total cholesterol level exceeding 5.2 mmol/L, or current treatment with lipid-lowering medications [20,21]. Diabetes mellitus was defined as an HbA1c of 6.5% or higher, or active use of antidiabetic drugs [20,21].

### 2.5. Plasma Protein Concentration Measurement

Blood samples were collected from patients, and plasma concentrations of 17 proteins related to angiogenesis were measured in duplicate using a commercially available LUMINEX assay (Bio-Techne, Minneapolis, MN, USA), following the manufacturer’s instructions [22]. The proteins selected for analysis were chosen based on their roles in angiogenic signaling pathways and documented relevance to cardiovascular pathology: HGF, angiopoietin-2, endothelin-1, endothelial growth factor (EGF), vascular endothelial growth factors (VEGF) A, C, and D, interleukin-8 (IL-8), granulocyte colony-stimulating factor (G-CSF), fibroblast growth factor (FGF) 1 and 2, leptin, endoglin, bone morphogenic protein 9 (BMP-9), heparin-binding epidermal growth factor-like growth factor (HB-EGF), follistatin, and placental growth factor (PLGF). The evaluation of various circulating proteins is intended to uncover new biomarker candidates for PAD. Prior to analysis, the MagPix system (Luminex Corp; Austin, TX, USA) [23] was calibrated using Fluidics Verification and Calibration bead kits (Luminex Corp; Austin, TX, USA) [24]. To minimize variability across assays, all measurements were performed on a single day. Intra- and inter-assay coefficients of variation were consistently kept under 10%. For each protein target, at least 50 beads were collected and analyzed using Luminex xPonent software version 4.3 (Luminex Corp; Austin, TX, USA) [25].

### 2.6. Outcomes

Patients returned for routine follow-up visits at one and two years after their initial assessment, with additional appointments scheduled as clinically indicated. The primary endpoint of the study was the incidence of MACE within the two-year follow-up period. MACE was defined as a composite of myocardial infarction (MI), stroke, or death, determined through direct follow-up. MI was diagnosed based on a rise and/or fall in cardiac troponin levels, with at least one value exceeding the 99th percentile upper reference limit, accompanied by one or more of the following criteria: (a) new ischemic changes on an electrocardiogram, (b) symptoms suggestive of myocardial ischemia, (c) the development of pathological Q waves, (d) imaging findings showing new loss of viable myocardium or regional wall motion abnormality in a pattern consistent with ischemia, or (e) angiographic or autopsy confirmation of coronary thrombus [26]. This definition aligns with recommendations from the American Heart Association, American College of Cardiology, European Society of Cardiology, and World Heart Federation [26]. Stroke was defined as death of brain, spinal cord, or retinal cells due to ischemia, determined by either (a) pathological, imaging, or other objective evidence of localized ischemic injury in a vascular territory, or (b) clinical symptoms consistent with focal ischemia lasting ≥24 h or leading to death, provided alternative causes were excluded [27]. This definition adheres to criteria set forth by the American Stroke Association and American Heart Association [27]. Death was defined as all-cause mortality. If patients have multiple events (e.g., MI and stroke), they are counted as having 1 composite MACE endpoint such that a single patient can have a maximum of 1 composite MACE outcome during the 2-year follow-up period.

### 2.7. Statistical Analysis

Baseline demographic and clinical outcome data were presented as means with standard deviations (SDs) for continuous variables, and as frequencies with corresponding percentages for categorical variables. To compare plasma protein concentrations between patients who did and did not experience MACE within two years, the Mann–Whitney U test was used. Proteins found to be differentially expressed between the two groups were further evaluated for their prognostic relevance. Cox proportional hazards models were applied to assess the association between these proteins and 2-year MACE, adjusting for relevant covariates including age, sex, hypertension, dyslipidemia, diabetes, smoking status (past or current), CHF, CAD, and prior stroke. Patients were stratified into low and high protein concentration groups based on the median concentration of each significant protein. Kaplan–Meier survival curves were used to assess MACE-free survival across these stratified groups, with statistical comparisons performed using Cox models adjusted for the same baseline variables. The objective of this analysis was to evaluate the prognostic significance of each protein, offering insight into how varying expression levels may influence MACE risk. A two-sided *p*-value < 0.05 was considered statistically significant. All statistical analyses were performed using SPSS version 23 (SPSS Inc., Chicago, IL, USA) [28].

## 3. Results

### 3.1. Patients

In the cohort, the average age was 69 years (SD ± 9), and 80 participants (32%) were female. As anticipated in a PAD population, the burden of cardiovascular comorbidities was substantial: 77% had hypertension, 82% had dyslipidemia, 42% had diabetes, 53% reported a history of smoking, and 30% were current smokers. Additionally, 6% had CHF, 42% had CAD, and 21% had experienced a prior stroke (Table 1). These results reflect a typical PAD population with an older age and a high prevalence of cardiovascular comorbidities.

### 3.2. Major Adverse Cardiovascular Events

Over the 2-year follow-up period, MACE was observed in 48 participants (19.8%). These included 37 cases of MI (15.3%), 13 strokes (5.4%), and 3 deaths (1.2%). Overall, there were 53 events that occurred in 48 patients, meaning that some patients suffered multiple events (Table 2). These results highlight the high rate of adverse cardiovascular events in the PAD population.

### 3.3. Plasma Concentrations of Angiogenesis-Related Proteins

Out of the 17 angiogenesis-related proteins evaluated, only two—HGF and angiopoietin-2—showed significantly higher levels in patients who experienced MACE within 2 years compared to those who did not: HGF (390.83 [SD 319.16] vs. 300.55 [SD 177.53] pg/mL, *p* < 0.001) and angiopoietin-2 (23.67 [SD 17.60] vs. 19.36 [SD 12.06] pg/mL, *p* = 0.020). No statistically significant differences were found for the remaining proteins (Table 3). These results demonstrate that HGF and angiopoietin-2 were the only two circulating proteins that were elevated in patients with 2-year MACE compared to those without.

### 3.4. Associations Between Angiogenesis-Related Proteins and MACE in PAD Patients

After controlling for baseline variables including sex, age, dyslipidemia, hypertension, smoking status (past or current), diabetes, CAD, CHF, and prior stroke, both HGF and angiopoietin-2 plasma levels remained independently associated with the occurrence of MACE at 2 years. Specifically, HGF showed an adjusted hazard ratio (HR) of 1.37 (95% CI: 1.14–1.64, *p* = 0.001), while angiopoietin-2 had an adjusted HR of 1.27 (95% CI: 1.04–1.55, *p* = 0.016) (Table 4). These results show that HGF and angiopoietin-2 were independently associated with 2-year MACE, highlighting their potential as prognostic biomarkers for systemic cardiovascular risk in patients with PAD.

### 3.5. Kaplan–Meier Analysis of Freedom from MACE in PAD Patients with High vs. Low Levels of HGF and Angiopoietin-2

**Figure 1:**
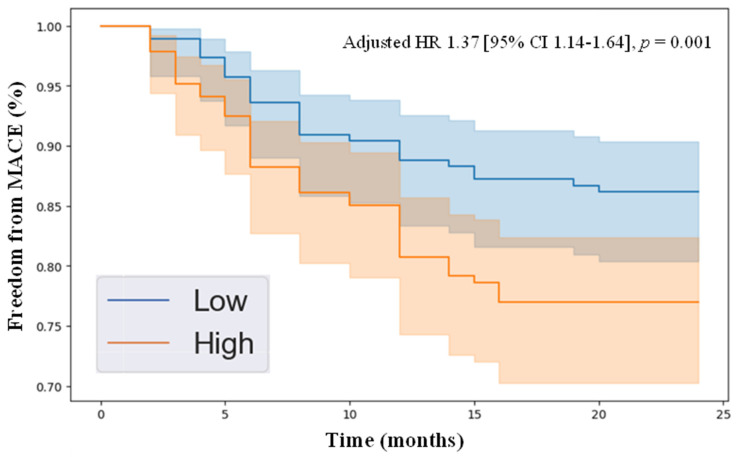
Kaplan–Meier survival analysis showing freedom from major adverse cardiovascular events in individuals with peripheral artery disease, stratified by high versus low HGF levels, determined by the median plasma concentration in the cohort of 317.9 pg/mL. The Cox proportional hazards model was applied, adjusting for variables including age, sex, hypertension, dyslipidemia, diabetes, current/past smoking, congestive heart failure, coronary artery disease, and previous stroke. Abbreviations: HR (hazard ratio), MACE (major adverse cardiovascular event), and CI (confidence interval).

**Figure 2:**
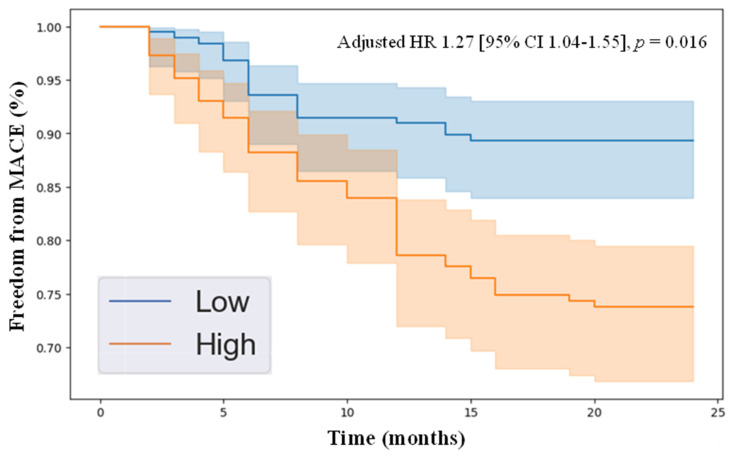
Kaplan–Meier survival analysis showing freedom from major adverse cardiovascular events in individuals with peripheral artery disease, stratified by high versus low angiopoietin-2 levels, determined by the median plasma concentration in the cohort of 20.2 pg/mL. The Cox proportional hazards model was applied, adjusting for variables including age, sex, hypertension, dyslipidemia, diabetes, current/past smoking, congestive heart failure, coronary artery disease, and previous stroke. Abbreviations: HR (hazard ratio), MACE (major adverse cardiovascular event), and CI (confidence interval).

Kaplan–Meier survival analysis demonstrated that individuals with plasma concentrations of HGF and angiopoietin-2 above the median in the cohort had significantly reduced freedom from MACE over the 2-year follow-up compared to those with protein levels below the median. Specifically, the adjusted hazard ratios were as follows: HGF (adjusted HR 1.37 [95% CI 1.14–1.64], *p* = 0.001, Figure 1) and angiopoietin-2 (adjusted HR 1.27 [95% CI 1.04–1.55], *p* = 0.016, Figure 2). These results highlight that patients with elevated levels of HGF and angiopoietin-2 were more likely to develop MACE over a 2-year follow-up period, demonstrating the clinical relevance of these novel biomarkers.

## 4. Discussion

### 4.1. Summary of Findings

In this study, we identified HGF and angiopoietin-2 as angiogenesis-related proteins independently associated with 2-year MACE in patients with PAD, suggesting their potential as prognostic biomarkers. Among the 17 proteins analyzed, only these two were significantly elevated in individuals who experienced 2-year MACE. After adjusting for baseline demographic and clinical characteristics, both remained independently predictive of 2-year MACE. Kaplan–Meier analysis further revealed that patients with higher median plasma levels of HGF or angiopoietin-2 had significantly lower freedom from MACE over two years. These findings underscore the potential clinical utility of these biomarkers in risk stratification and in guiding cardiovascular risk reduction strategies in patients with PAD.

### 4.2. Comparison to Existing Literature

Previous studies have emphasized the role of angiogenesis-related proteins in cardiovascular pathophysiology. Guo et al. (2018) highlighted their diagnostic and therapeutic implications in CAD, noting that myocardial ischemia can stimulate angiogenesis during both acute and chronic phases within experimental models [29]. In the PAD population, a 2017 Cochrane Systematic Review evaluated 20 clinical trials that examined the impact of various angiogenic proteins, including HGF, on cardiovascular outcomes [13]. While no significant effect was found on major amputation or mortality, some trials reported improvements in hemodynamic parameters, ulcer healing, and rest pain [13]. However, the overall evidence quality was rated as low due to methodological limitations [13]. Unlike the therapeutic focus of these trials, our study evaluated the prognostic value of angiogenesis-related proteins for MACE risk in PAD patients. We found that HGF and angiopoietin-2 were significantly associated with an increased risk of 2-year MACE, suggesting their potential utility as biomarkers to identify individuals at elevated risk of systemic vascular complications. Supporting this finding, Garg et al. (2020) reported that elevated HGF levels were linked to a greater risk of PAD development [30]. Given these associations, our findings may inform a more proactive treatment approach in clinical practice. Patients with high HGF concentrations—who are at an elevated MACE risk—may benefit from intensified management strategies, including advanced pharmacologic therapy, gene therapy, or early surgical intervention [31].

Similar to HGF, angiopoietin-2 plays a central role in angiogenesis and inflammatory regulation, both of which are implicated in PAD, CAD, and CVD [32]. In the vascular surgery literature, Golledge et al. (2013) observed higher angiopoietin-2 levels in patients with abdominal aortic aneurysms, linking them to increased cardiovascular mortality [32]. Patel et al. further showed that this protein predicted MI in individuals with hypertension [33]. Peplinski et al. (2021) showed that angiopoietin-2 was associated with heart failure or death among adults without cardiovascular disease at baseline and with disease severity in individuals with existing heart failure within the Multi-Ethnic Study of Atherosclerosis [34]. Our findings align with this growing body of evidence. We demonstrated a strong association between angiopoietin-2 and 2-year MACE in PAD, reinforcing its potential as a biomarker for systemic vascular risk. Our group recently showed that angiogenesis-related proteins were associated with MALE in patients with PAD [9]. By extending these findings to cardiovascular outcomes, our study highlights the broader systemic relevance of HGF and angiopoietin-2. This reinforces the need for further mechanistic studies exploring their role in the intersection of PAD, CAD, and CVD. Ultimately, this could lead to improved risk stratification and the development of novel targeted therapies to improve systemic cardiovascular outcomes in the PAD population.

### 4.3. Explanation of Findings

Several mechanistic explanations may account for the observed associations of HGF and angiopoietin-2 with elevated MACE risk in patients with PAD. Initially characterized as a mitogen for hepatocytes, HGF is a multifunctional growth factor essential for tissue repair and regeneration [35]. It is synthesized as a heterodimer composed of 69 kDa alpha and 34 kDa beta chains, primarily stromal cells [36]. HGF plays key roles in morphogenesis, cell proliferation, and angiogenesis, largely through activation of its receptor, c-Met, via phosphorylation [35]. Beyond its regenerative role in hepatic tissue, HGF has been shown to contribute to endothelial repair and the formation of collateral blood vessels, improving perfusion in ischemic tissues affected by atherosclerosis [37]. Given these insights, it is plausible that elevated circulating HGF levels in PAD patients may reflect a compensatory—but insufficient—response to underlying vascular injury and impaired angiogenesis [38]. This could hinder adequate collateral formation across multiple vascular beds, compromising perfusion to critical organs such as the heart and brain, ultimately leading to increased MACE incidence [38].

Angiopoietin-2, another protein of interest in our study, is a well-characterized member of the angiopoietin family, known for its regulatory functions in vessel development and stability [39]. Structurally, it consists of a 496-amino-acid polypeptide chain and exhibits a receptor-binding domain formed by a central antiparallel β-sheet surrounded by alpha helices, enabling interaction with the Tie2 receptor and integrins [40]. Mainly produced in endothelial cells and stored within Weibel-Palade bodies, angiopoietin-2 is rapidly secreted in response to hypoxic or inflammatory stimuli [41]. Its release disrupts endothelial junctions, promotes extracellular matrix degradation, and activates adhesion molecules that facilitate leukocyte migration, thereby amplifying vascular inflammation and permeability [12]. These disruptions collectively contribute to vascular dysfunction and disease progression [12]. Lee et al. (2018) further demonstrated the deleterious effects of angiopoietin-2 in a murine model of MI, where it was upregulated in endothelial cells at the infarct border zone during both acute and chronic phases of cardiac injury [42]. In the acute setting, it facilitated pericyte loss, vascular leakiness, neutrophil infiltration, and degradation of vascular structures, while during chronic remodeling, it promoted ongoing inflammation and maladaptive vascular remodeling via endothelial and macrophage signaling pathways [42]. Altogether, these findings explain the biological underpinnings that link elevated levels of HGF and angiopoietin-2 to cardiovascular events in PAD patients. Their involvement in key processes such as impaired angiogenesis, vascular remodeling, and inflammation underscores their potential utility as prognostic biomarkers for MACE in the PAD population.

### 4.4. Implications

Our findings offer meaningful insights with direct clinical relevance for the management of patients with PAD. By measuring plasma concentrations of HGF and angiopoietin-2, clinicians can more accurately stratify PAD patients based on their risk of MACE. This approach is especially beneficial in family medicine, where early identification of high-risk individuals allows for more targeted care pathways [43]. General practitioners can adopt this biomarker-based strategy by integrating HGF and angiopoietin-2 testing into routine assessments of patients with PAD [43]. Those identified as having a heightened risk of MACE could be promptly referred to a multidisciplinary team involving cardiologists, vascular specialists, and neurologists for comprehensive evaluation and management [44]. Conversely, patients categorized as low risk could remain under the care of their primary physician, with a focus on optimizing cardiovascular risk through established measures, including antiplatelet therapy, statin use, and lifestyle interventions [45]. For patients referred to specialty care, our findings support the development of risk-adapted treatment strategies. For example, incorporating low-dose rivaroxaban alongside aspirin has demonstrated benefit in reducing cardiovascular events in individuals with stable PAD or CAD [46]. Additionally, advanced imaging such as coronary or cerebral angiography may be warranted in high-risk patients to detect and treat critical atherosclerotic lesions before they result in clinical events [47]. In summary, the integration of angiogenic biomarkers into PAD care has the potential to significantly enhance patient outcomes by supporting more personalized risk assessment, refining referral practices, and enabling more tailored treatment decisions [48].

### 4.5. Limitations

There are several limitations to this study that should be considered when interpreting the findings. First, as the investigation was carried out at a single academic center, the applicability of our findings to broader and more diverse patient populations requires further study. Validation through multicenter studies will be essential to confirm the generalizability of the results. Second, the analysis was limited to a two-year follow-up period. Given the chronic and progressive nature of PAD, CAD, and CVD, longer-term follow-up is necessary to better understand the sustained prognostic value of HGF and angiopoietin-2. Third, this study was not sufficiently powered to explore associations between the biomarkers and individual components of MACE. Future research involving larger cohorts and a greater number of clinical events is needed to more precisely evaluate the predictive capacity of these proteins for specific MACE outcomes. Lastly, the measurement of HGF and angiopoietin-2 currently occurs predominantly within research environments. Further translational studies and implementation research are required to evaluate the practicality, cost-effectiveness, and clinical integration of these biomarkers into routine care for patients with PAD.

## 5. Conclusions

This study identified HGF and angiopoietin-2 as angiogenesis-related proteins that are independently associated with 2-year MACE in patients with PAD, suggesting their potential as biomarkers for systemic atherosclerosis. Specifically, PAD patients with elevated plasma concentrations of HGF and angiopoietin-2 are more likely to develop MACE over a 2-year follow-up period. The predictive value of these novel biomarkers could support more precise risk stratification and personalized management strategies in clinical settings. Identifying high-risk individuals enables earlier, more aggressive intervention—such as referrals to multidisciplinary specialists and intensified medical therapy—aimed at preventing MI and stroke, which remain the leading causes of mortality in the PAD population.

## Figures and Tables

**Table 1:** Baseline characteristics.

	Patients with PAD (*n* = 250)
Age, mean ± SD, years	69 ± 9
Female sex	80 (32%)
Hypertension	193 (77%)
Dyslipidemia	204 (82%)
Diabetes	104 (42%)
Past smoker	131 (53%)
Current smoker	75 (30%)
Congestive heart failure	14 (6%)
Coronary artery disease	105 (42%)
Previous stroke	53 (21%)

Unless otherwise specified, data are presented as counts (percentages). Abbreviations used: SD—standard deviation; PAD—peripheral artery disease.

**Table 2:** Outcomes over 2 years of follow-up.

	Patients with PAD (*n* = 250)
Major adverse cardiovascular event	48 (19.8%)
Myocardial infarction	37 (15.3%)
Stroke	13 (5.4%)
Death	3 (1.2%)

Data are presented as counts (percentages). Abbreviation used: PAD—peripheral artery disease.

**Table 3:** Plasma protein concentrations.

	No MACE (*n* = 202)	MACE (*n* = 48)	*p*-Value
**HGF**	**300.55 (177.53)**	**390.83 (319.16)**	**<0.001**
**Angiopoietin-2**	**19.36 (12.06)**	**23.67 (17.60)**	**0.020**
BMP-9	160.48 (178.29)	122.13 (110.64)	0.091
Leptin	27.28 (30.00)	33.77 (46.03)	0.153
Follistatin	673.24 (387.61)	741.93 (339.89)	0.181
VEGF-D	389.16 (741.60)	269.72 (559.28)	0.210
PLGF	3.99 (5.66)	3.12 (3.42)	0.233
IL-8	6.52 (6.08)	7.42 (5.70)	0.266
HB-EGF	41.30 (35.65)	46.27 (42.09)	0.322
VEGF-A	125.10 (158.39)	135.54 (144.46)	0.621
EGF	64.28 (107.36)	59.00 (59.17)	0.695
G-CSF	123.49 (144.57)	130.74 (169.76)	0.722
Endothelin-1	4.90 (7.56)	5.15 (8.25)	0.819
FGF-1	10.97 (7.31)	10.76 (7.68)	0.830
Endoglin	1422.07 (727.86)	1403.68 (764.04)	0.855
FGF-2	111.37 (98.78)	109.28 (78.08)	0.874
VEGF-C	980.10 (637.90)	989.32 (593.37)	0.912

Protein concentrations are reported as the mean (standard deviation), pg/mL. Rows in bold are statistically significant (*p* < 0.05). Abbreviations: hepatocyte growth factor (HGF), interleukin-8 (IL-8), endothelial growth factor (EGF), fibroblast growth factor (FGF), vascular endothelial growth factor (VEGF), leptin, granulocyte colony-stimulating factor (G-CSF), endoglin, bone morphogenic protein 9 (BMP-9), follistatin, heparin-binding epidermal growth factor-like growth factor (HB-EGF), placental growth factor (PLGF), and MACE (major adverse cardiovascular event).

**Table 4:** Adjusted hazard ratios for the associations between HGF and angiopoietin-2 and MACE over 2 years of follow-up.

	Adjusted Hazard Ratio	Lower 95% CI	Upper 95% CI	*p*-Value
HGF	1.37	1.14	1.64	**0.001**
Angiopoietin-2	1.27	1.04	1.55	**0.016**

Adjusted for age, sex, hypertension, dyslipidemia, diabetes, past/current smoking, congestive heart failure, coronary artery disease, and previous stroke. Hazard ratios were determined for each 1 pg/mL increase in plasma protein concentration. Rows in bold are statistically significant (*p* < 0.05). Abbreviations: hepatocyte growth factor (HGF); CI (confidence interval).

## Data Availability

The original contributions presented in this study are included in the article; further inquiries can be directed to the corresponding author.
